# 

**Published:** 1881-11

**Authors:** 


### CONTENTS.

### COMMUNICATIONS.

Address .... 443
Anæsthesia—Nitrous Oxide .... 458
The Trials of the Profession .... 466

### SELECTIONS.

Give Your Titles .... 471
A Hitherto Undescribed Disease of Childhood .... 471
The Medicinal Use of the Tomato .... 472
Whisky and Sunstroke .... 473
A Plea for General Education of Subjects of Hygiene .... 473
Vitality of Infants .... 474
Hot Water for the Heart .... 475
Death from Anæsthetics .... 475
Pregnancy and Caries of the Teeth .... 476
Inoculation of Saliva .... 476
Analysis of the Human Breath .... 477
Digestive Ferment in the Fig .... 477
Paresi's Hæmostatic Collodion .... 478
Origin of the Nervous System .... 478
Elevation of the Epiglottis .... 478
Syrup of Chloral .... 479
Maternal Impressions .... 479
Pain Cured by Hypodermic Injections of Cold Water .... 480
Septicæmia Resulting from a Leech Bite .... 481
Correspondence .... 481

### EDITORIAL.

Nitrous Oxide .... 483
A New Journal .... 484
Examiners' Meeting .... 485
Ohio State Dental Society .... 485
Removal .... 486
The Ohio State Dental Society .... 486

